# Financial toxicity of breast cancer over the last 30 years: A bibliometrics study and visualization analysis via CiteSpace

**DOI:** 10.1097/MD.0000000000033239

**Published:** 2023-03-24

**Authors:** Hui Cheng, Lu Lin, Tingting Liu, Shaotong Wang, Yueyue Zhang, Li Tian

**Affiliations:** a The First Affiliated Hospital of Soochow University, Suzhou, China; b School of Nursing, Medical College of Soochow University, Suzhou, China.

**Keywords:** bibliometrics, breast cancer, financial toxicity, visualization analysis

## Abstract

This literature on financial toxicity (FT) of breast cancer aimed to identify the leading countries, institutions, key researchers, influential references, top journals, research hotspots, and frontiers in the field. Published articles on FT in breast cancer patients were systematically retrieved and screened from the Web of Science databases from inception to March 28, 2022. The CiteSpace software was used to generate knowledge maps to analyze bibliometric characteristics in FT research on breast cancer patients. A total of 615 publications were included, with a year-on-year increase in the number of publications. A total of 591 authors conducted research on the FT in breast cancer patients, with Yabroff KR being the most prolific author. The US was the absolute leader in this field, with almost all major research institutions and authors located in the US. *Supportive Care in Cancer* was the most productive journal, and the *Journal of Clinical Oncology* was the most co-cited journal. The keywords representing the research hotspots were “quality of life,” “care,” “cost,” etc. Keywords burst detection indicated that “financial toxicity,” “survivors,” “impact,” “burden,” “income,” and “experience” have become the new research frontiers in the last 5 years. There is an overall upward trend in the research on FT of breast cancer over the last 30 years, which has important and ongoing research value. There is still a paucity of relevant research and more collaboration between authors, institutions, and countries is needed in the future to identify future research directions.

## 1. Introduction

In 2020, the International Agency for Research on Cancer released the latest global cancer update with an estimated 19.3 million new cancer cases worldwide, including an estimated 2.3 million new cases of female breast cancer, accounting for 11.7% of all cancer cases and surpassing lung cancer as the leading cause of global cancer incidence.^[1]^ With the further development of anti-tumor therapy, new anti-cancer drugs, genetic testing, and other treatments have been continuously developed and improved. While improving the therapeutic effect and prolonging the survival of patients, the high cost of medical treatment imposes a heavy financial burden on patients.^[2]^ According to statistics, the medical expenses for cancer in the US increased by approximately $53 billion from 2010 to 2020, an increase of nearly 27%.^[3]^ The average expenditure of adult cancer patients is estimated to be $16,346, 4 times that of non-cancer patients.^[4]^ Financial toxicity (FT) is common among women with breast cancer due to the long-term nature of the treatment, the accumulation of treatment-related costs, and the inability to continue working while undergoing treatment and recovery.^[5]^ Although most breast cancer patients have insurance, there are still a majority of patients with FT.^[6]^ A study has found that 12 to 62% of cancer survivors report being in debt because of their treatment, 47 to 49% of survivors report experiencing some form of financial hardship, and 4 to 45% of survivors do not adhere to the recommended prescription drugs due to the cost of treatment.^[7]^ FT, proposed by American scholar S. Yousuf Zafar in 2013, refers to the subjective and objective financial distress of patients caused by cancer treatment and ongoing costs.^[8]^ Currently, FT has been listed as one of the potential adverse reactions in the treatment of malignant tumors.^[9]^ FT is a wide-ranging term that encompasses the costs incurred by patients following a cancer diagnosis, which has an impact on personal and family budgets.^[10]^ There are changes in activities of daily living due to the need for a special diet, escorting, work absenteeism and expenses associated with hiring caregivers, among others. The treatment, whether covered by social security or not, generates costs with tests and/or adjunctive medications, which can financially burden patients and families.^[10]^ A meta-analysis showed that compared with cancer patients with a lower financial burden, those with a higher financial burden were approximately twice as likely to have non-adherence to cancer medication and worse overall physical, mental, emotional, and social functioning and well-being.^[11]^ Because of this financial instability, negative outcomes can be seen on physical, mental, emotional, and economic levels.^[12]^ A systematic review revealed a positive correlation between FT and psychological symptoms.^[13]^ FT can increase the risk of depression,^[14]^ reduce the quality of life of patients, pose a threat to their health and life, and hinder the recovery of their social functions.^[15]^ With an ever-increasing prevalence of breast cancer, which is the most common malignant cancer among women worldwide in 140 of the 184 countries,^[16]^ it is imperative to understand the research status of FT in relation to this disease.

Visualization analysis has become a widely used method for analyzing correlations in massive data in recent years. It uses software to conduct correlation analysis of data and convert the results into a visual atlas, allowing for a more intuitive understanding of relevant information and making it easier to uncover the patterns hidden in big data and to quickly integrate effective information.^[17]^ Based on literature search, current studies and systematic reviews have summarized the evidence for the measures used to quantify FT,^[18]^ the risk factors for FT,^[11]^ and the prevalence and health-related consequences of FT in cancer patients.^[19]^ However, there is a lack of bibliometric and visual studies on the current status of FT research and research hotspots in breast cancer patients. Therefore, the purpose of this study was to analyze the literature on the FT in breast cancer patients using bibliometrics and visualization analysis to understand the current status of research and publication trends, analyze the collaboration among academic organizations, identify current research hotspots, and predict future research trends, to provide a reference for future research.

## 2. Methods

### 2.1. Search strategies

We searched the Web of Science Core Collection (WoSCC) for articles on FT in breast cancer patients from the inception of the database to March 28, 2022, using the following conditions: breast cancer and FT (search terms), English (language), article and review (type of literature). The relevance of the results was checked. The detailed search strategies are shown in the Appendix.

### 2.2. Inclusion and exclusion criteria

Inclusion criteria: original peer-reviewed articles or reviews.

Exclusion criteria: conference abstracts or errata documents; unpublished articles; duplicate publications; irrelevant articles.

### 2.3. Analysis tool

CiteSpace is a visualization software for bibliometric analysis developed by Professor Chaomei Chen based on the Java platform.^[20]^ It is an interactive analysis tool that enables visualization tasks in scientific mapping by combining bibliometrics, visualization analysis, and data mining algorithms.^[20]^ CiteSpace provides a variety of functional options for bibliometric research, including collaborative network analysis, co-citation analysis, and co-occurrence analysis, and can generate visual maps. By generating a series of visual knowledge maps, CiteSpace explores the current state of research, hotspots, frontiers, and evolution processes in a certain scientific field, reveals the research directions, research stages, and frontier characteristics of institutions and authors, and finally determines the development trend of this field.

The software version used in this study is 5.8 R3 (64-bit). The parameters of CiteSpace were as follows: time slicing (1994.1–2022.4), years per slice (1), term source (all selection), node type (choose one at a time), selection criteria (50), pruning (pathfinder, pruning sliced Networks), and the others were in default setting. When the default number of CiteSpace network nodes is >350, the centrality calculation function will be disabled. The “Computing Node centrality” function in the node menu needs to be manually enabled, and the nodes and links are used to generate visual knowledge maps.

### 2.4. Bibliometrics and visualization analysis

The database used in this study was Web of Science, and the articles were retrieved from the WoSCC, including SCI-Expanded, SSCI, CCR-Expanded, and Index Chemicus. The Web of Science database contains a large number of multidisciplinary, high-impact, international, and comprehensive academic journals, which provide a better knowledge map when using CiteSpace for visualization analysis.^[21,22]^ As the database is updated daily, the literature included in WoSCC was retrieved within 1 day on March 28, 2022, to avoid bias. The document data were saved in the form of complete records, the cited references were saved in plain text format. The data were then imported into CiteSpace for data format conversion, deduplication, and visualization before the results were presented bibliometrically and visually.^[23]^

## 3. Results

### 3.1. Quantity and trend analysis of published papers

**Figure 1.**
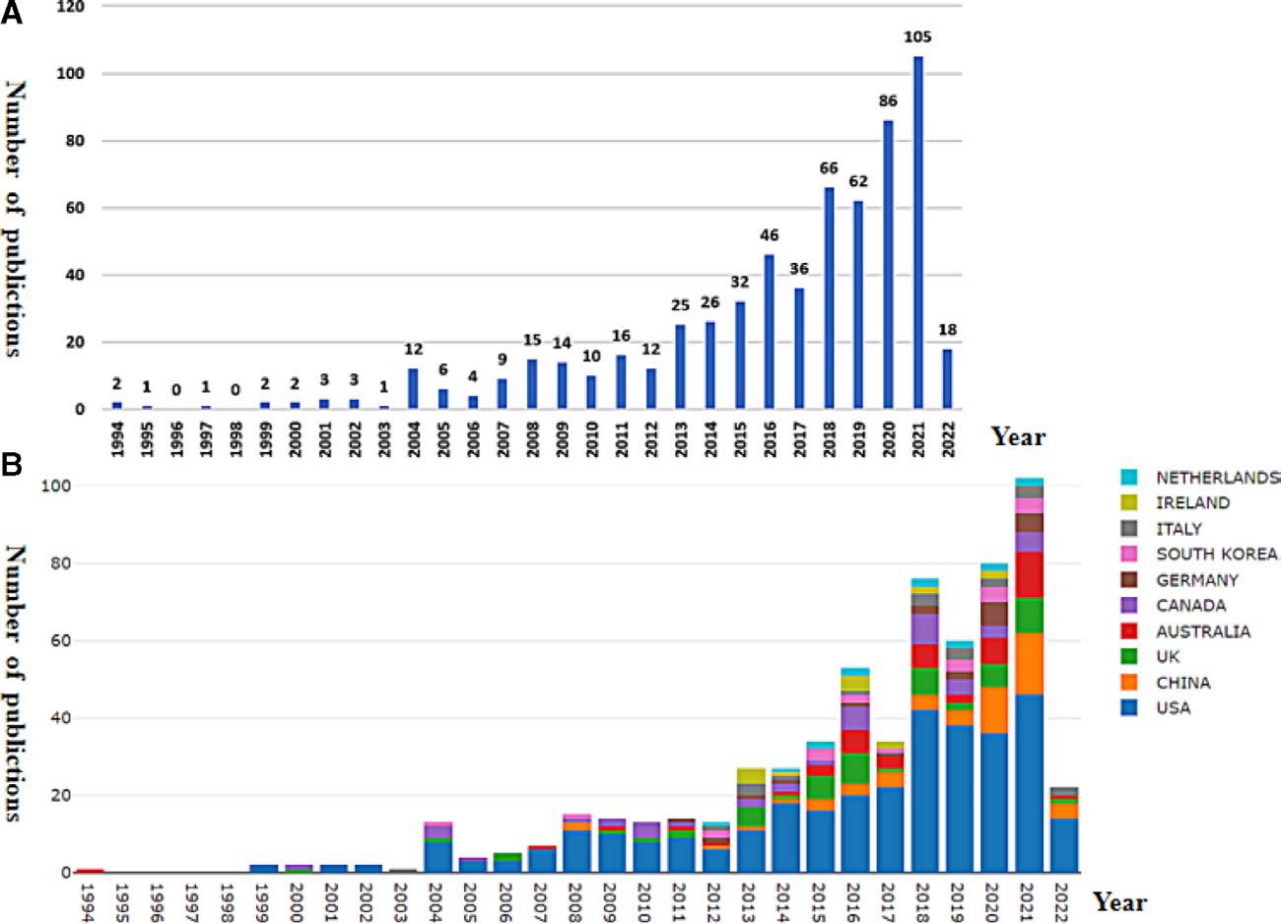
(A) Annual trend of publications. (B) Number of annual publications and growth trends of the top 10 countries/regions.

A total of 615 articles were included in this study, and the actual number of articles published each year was calculated using the bibliometrics online analysis platform (http://bibliometric.com/). In the early stage (1994–2010), the number of papers per year was <10, except in 2004, 2008, and 2009 (Fig. 1A). In 2021, the number of papers published on the topic peaked, showing that the FT in breast cancer has become a research hotspot and attracted the attention of researchers worldwide.

In order to identify countries/regions leading research in this area, further analysis of the literature from different countries/regions was conducted. The bar chart (Fig. 1B) shows the top 10 countries/regions in the total number of published articles in the past 30 years. The results show that the number of publications is still growing steadily, and the US is identified as the founder of this field. It has also been found that annual publications in China are growing rapidly.

### 3.2. Analysis of international/interregional and interinstitutional cooperation

**Figure 2.**
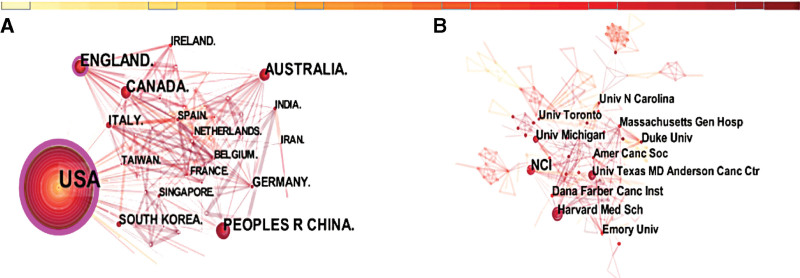
(A) The network of co-country (with a threshold of 10 publications). (B) The network of co-institution (with a threshold of 10 publications).

To identify interinstitutional collaborations to conduct such studies, international/interregional and interinstitutional analyses were performed using CiteSpace. The size of concentric circles indicates the number of articles published, and institutions that publish more articles tend to have larger concentric circles. The connection between the 2 circles means they published together, and the thickness of the lines indicates the strength of their collaboration. The results show that 86 countries have already established partnerships, with 286 links to each other. Centrality is also known as intermediate centrality, and nodes with high centrality (>0.1) are generally regarded as turning points or key points in this field.^[24]^ The US had the highest centrality (0.64) and the best partnerships in this area, followed by the UK (0.22) and Australia (0.1). However, China had less centrality (0.01) and less international cooperation than the US (Fig. 2A).

The interinstitutional cooperation network diagram (Fig. 2B) shows 447 nodes and 818 links. Institutions located in the US accounted for the vast majority of the total. The top 5 institutions, all in the US, were the National Cancer Center (25 articles), Harvard Medical School (16 articles), the University of Michigan (16 articles), the Dana-Farber Cancer Institute (15 articles), and Duke University (14 articles).

### 3.3. Author and author co-citation analysis

**Figure 3.**
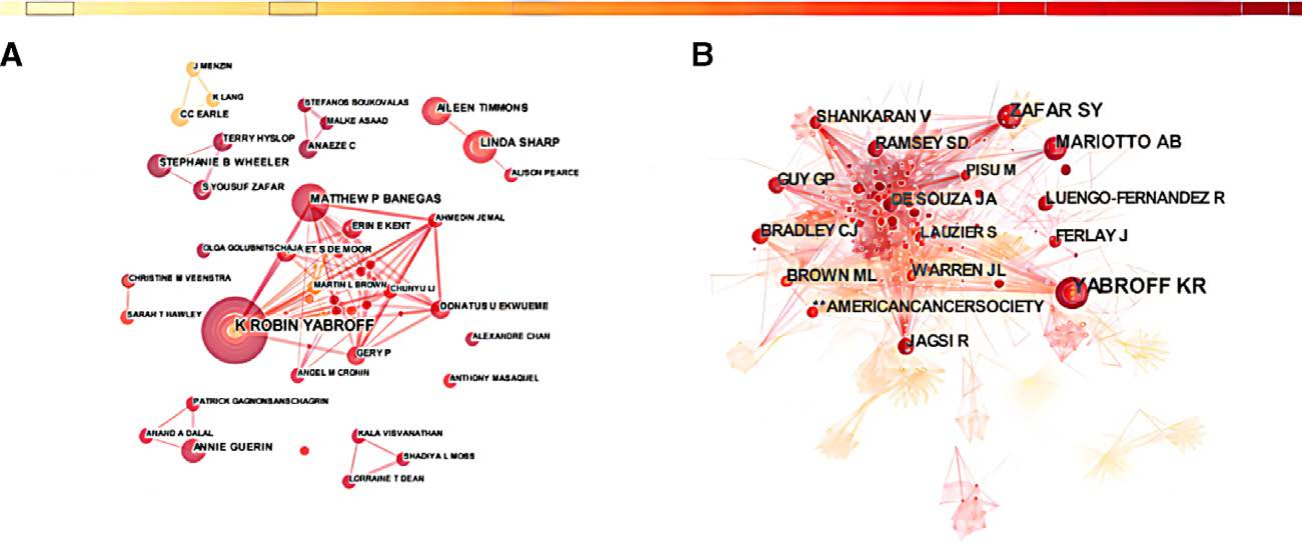
(A) The network of co-authorship (with a threshold of 3 publications). (B) The network of author co-citation (with a threshold of 35 publications).

The visualization atlas of 591 nodes and 873 links formed by the combined coauthor network is shown in Figure 3A. The co-authorship network shows the prolific authors and their cooperation. The most productive author was Yabroff KR with 19 articles, followed by Sharp L (11 articles) and Guy GP (10 articles), with no Chinese authors in the top 10. Although many of the authors have published relevant articles, there is little collaboration between them. In addition, the relatively low centrality of the authors indicates that more high-quality and large-scale collaborations are needed in the future (Table 1).

**Table 1:** The top 10 most productive authors.

N	Author	Frequency	Percentage (%)	Country
1	Yabroff KR	19	3.089	US
2	Sharp L	11	1.789	UK
3	Guy GP	10	1.626	US
4	Banegas MP	9	1.463	US
5	Ekwueme DU	9	1.463	US
6	Pisu M	9	1.463	US
7	Azuero A	8	1.301	US
8	Offodile AC	8	1.301	US
9	Wheeler SB	8	1.301	US
10	Hawley ST	7	1.138	US

A co-citation relationship between authors is established when 2 (or more) authors are simultaneously cited in 1 or more subsequent papers. An analysis of an author’s co-citation network provides a clear picture of the core authors and their contributions to a field, the strength of which indicates the level of author involvement. The most co-cited authors were Yabroff KR (115 citations), followed by Zafar SY (88 citations), Mariotto AB (63 citations), Bradley CJ (54 citations), and Jagsi R (53 citations). In addition, Brown ML (0.17), Baker MS (0.13), and Yabroff KR (0.13) were the authors of high centrality (Fig. 3B).

### 3.4. Journal and journal co-cited analysis

Journal analysis and co-cited journal analysis can provide important information to understand journals with a high volume of publications. It is not only beneficial for researchers to timely discover the latest research trends in the field and be selective when submitting manuscripts, thus improving the publication rate, but also conducive to finding suitable collaborating institutions and promoting domestic and international academic exchanges,^[25]^ thus improving the overall research level of FT of breast cancer. The WoSCC search revealed that the 615 articles included in the current analysis were published in 298 different journals, and the 10 journals with the most publications were identified through bibliometrics online analysis methods (Table 2).

**Table 2:** The top 10 journals contributing to published articles on financial toxicity of breast cancer.

Journal	IF (JCR 2021)	Quartile in category (JCR)	Article counts	Percentage (%)
*SUPPORTIVE CARE IN CANCER*	3.603	Q2	34	5.528
*BREAST CANCER RESEARCH AND TREATMENT*	4.872	Q2	22	3.577
*CANCER*	6.860	Q1	22	3.577
*JOURNAL OF CLINICAL ONCOLOGY*	44.544	Q1	13	2.114
*PLOS ONE*	3.240	Q2	12	1.951
*PSYCHO-ONCOLOGY*	3.894	Q2	12	1.951
*JNCI JOURNAL OF THE NATIONAL CANCER INSTITUTE*	11.577	Q1	11	1.789
*BMC CANCER*	4.430	Q3	10	1.626
*BMC HEALTH SERVICES RESEARCH*	2.655	Q3	9	1.463
*ONCOLOGIST*	5.462	Q2	9	1.463

This JCR division is Thomson Reuters division that is journals of the same discipline are divided into Q1 (top 0–25%), Q2 (top 25–50%), Q3 (top 50–75%), and Q4 (top 75–100%) on average according to the influence factors.

IF = impact factor.

**Figure 4.**
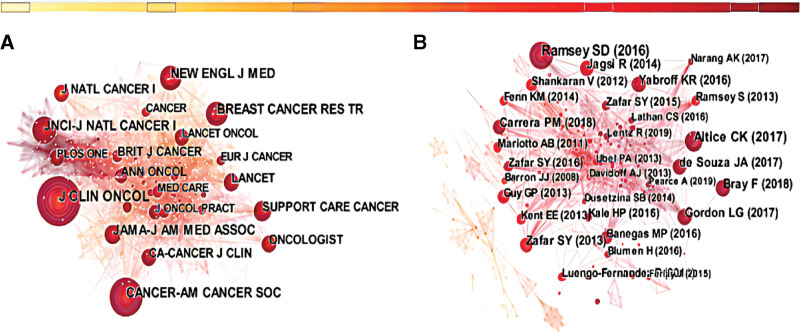
(A) The network of journal co-citation (with a threshold of 120 publications). (B) The network of co-cited references (with a threshold of 10 publications).

The analysis of the cited journals showed that the most cited journals were *Journal of Clinical Oncology* (393 citations), *Cancer* (306 citations), *JNCI Journal of the National Cancer Institute* (244 citations), *New England Journal of Medicine* (237 citations), and *Breast Cancer Research and Treatment* (224 citations) (Fig. 4A).

### 3.5. References and co-cited references analysis

Figure 4B shows the top references with a high frequency of co-citations. The most co-cited reference published by Ramsey SD^[7]^ in 2016 elaborated on the serious financial distress faced by cancer patients after diagnosis, which may be related to cancer mortality. The second was a systematic review of the financial difficulties of cancer patients published by Altice CK^[26]^ in 2016. The top 10 cited references out of the 615 included articles were listed in Table 3.

**Table 3:** The top 10 high-cited references in 615 articles on financial toxicity of breast cancer.

Title	Author	Journal	Year	Citation
Family caregiver burden: results of a longitudinal study of breast cancer patients and their principal caregivers	Grunfeld, E	*Canadian Medical Association Journal*	2004	584
The financial toxicity of cancer treatment: a pilot study assessing out-of-pocket expenses and the insured cancer patient’s experience	Zafar, S.	*Oncologist*	2013	536
Cancer-related fatigue: the scale of the problem	Hofman, Maarten	*Oncologist*	2007	523
Economic burden of cancer across the European Union: a population-based cost analysis	Luengo-Fernandez, Ramon	*LANCET Oncology*	2013	513
Cost of care for elderly cancer patients in the United States	Yabroff, KR	*JNCI Journal of The National Cancer Institute*	2008	493
Burden of illness in cancer survivors: findings from a population-based national sample	Yabroff, KR	*Jnci-Journal of The National Cancer Institute*	2004	417
Incidence, treatment costs, and complications of lymphedema after breast cancer among women of working age: a 2-year follow-up study	Shih, Ya-Chen Tina	*Journal of Clinical Oncology*	2009	299
Economic burden of cancer in the United States: estimates, projections, and future research	Yabroff, KR	*Cancer Epidemiology Biomarkers & Prevention*	2011	289
Financial hardships experienced by cancer survivors: a systematic review	Altice, Cheryl K	*JNCI-Journal of The National Cancer Institute*	2017	284
Financial hardship associated with cancer in the United States: findings from a population-based sample of adult cancer survivors	Yabroff, KR	*Journal of Clinical Oncology*	2016	235

### 3.6. Analysis of keywords

**Figure 5.**
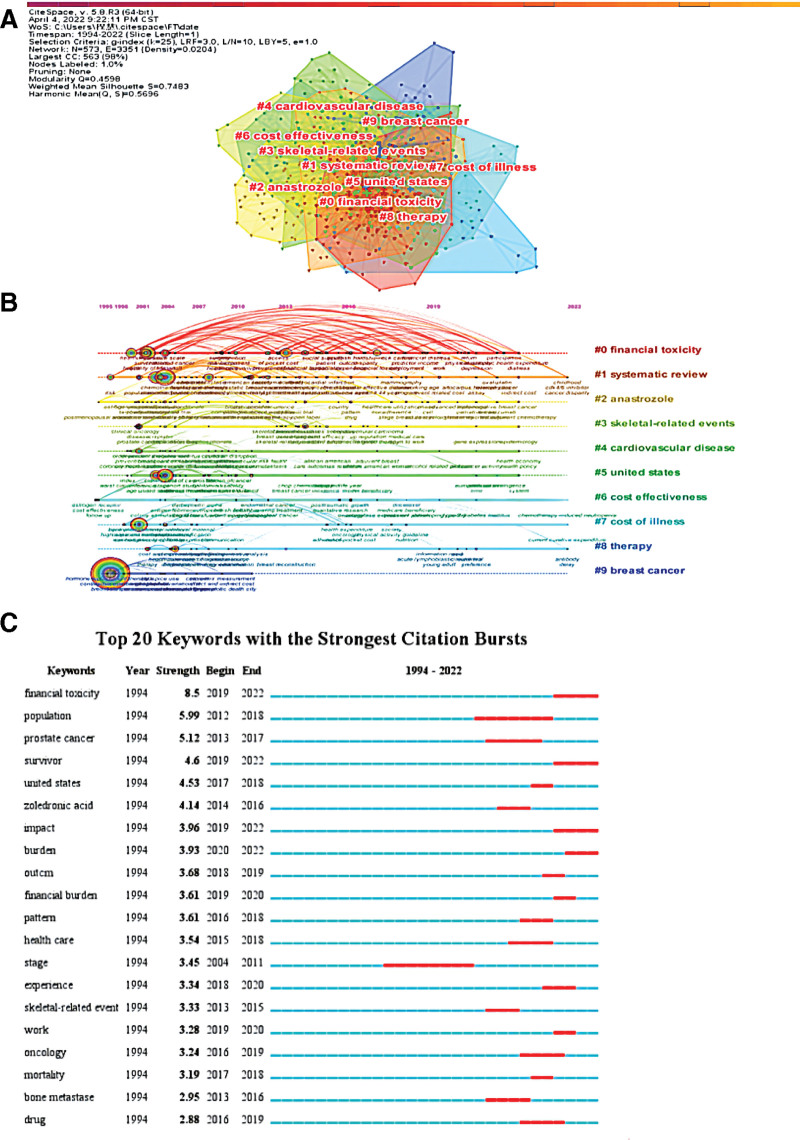
(A) The network of co-occurring keywords clusters. (B) Timeline view of the top 10 largest clusters of keywords, Right side = cluster labels. (C) Top 20 keywords with the strongest citation bursts.

Keywords are a summary of the research topic of an article, and the main research directions of a certain discipline can be understood by analyzing the frequency of keywords.^[27]^ A total of 573 keyword nodes and 2330 keyword lines were obtained, with a density of 0.0142. Word frequency analysis is a measurement method to analyze the research hotspots by counting the frequency of keywords or subject terms in the literature.^[28]^ Excluding the search terms, the top 5 keywords in frequency in this study were “care” (104) “quality of life” (90) “cost” (80) “women” (78), and “impact” (62), as shown in Table 4. Centrality analysis is a measure of the importance of nodes in the network. The greater its value, the higher the representation and attention of nodes in the network.^[24]^ Among the high-frequency keywords, “quality of life” (0.14) had the highest centrality, indicating the large number and influence of related studies conducted with this keyword. Keywords cluster analysis was performed on the retrieved literature on FT in breast cancer patients to explore research hotspots in this field. The calculated *Q* value (Q) and silhouette value (S) were indicators representing the modularity and homogeneity of the cluster network, respectively. A Q > 0.3 identified the cluster structure as significant, and an S > 0.5 or > 0.7 indicated that the clustering result was reasonable or highly credible, respectively.^[29]^ In the cluster map of this study, Q = 0.4598 and S = 0.7483, indicating significant cluster structure and reasonable results. The cluster labels and main keywords are shown in Figure 5A.

**Table 4:** The top 10 keywords in terms of frequency and centrality.

N	Frequency	Centrality	Year	Keyword
1	104	0.12	2001	care
2	90	0.14	2002	quality of life
3	80	0.09	2004	cost
4	78	0.11	2003	women
5	62	0.03	2013	impact
6	61	0.03	2002	survivor
7	57	0.06	2003	United States
8	54	0.07	2000	health
9	48	0.03	2004	burden
10	43	0.12	2004	chemotherapy

The timeline distribution of keywords not only reveals the specific keywords included in each cluster but also shows the beginning and ending time nodes of each cluster theme, thus helping to sort out the development path of FT research in breast cancer patients (Fig. 5B).

Keywords burst detection are used to determine the development trend of the research field based on the trend of word frequency changes of the subject terms. Burst detection reveals the sudden changes in terms or references within a specific period of time. Words with high burst intensity represent frontier issues of research in the corresponding era, thus identifying emerging research trends.^[30]^ “Financial toxicity,” “survivor,” “impact,” “burden,” “outcome,” and “experience” are the newly emerging terms in the last 5 years (Fig. 5C).

## 4. Discussions

The number of articles published is an important indicator of scientific research activities, which can reflect, to a certain extent, the development of the discipline and the attention of society.^[31]^ This study used CiteSpace as a quantitative assessment tool to examine the literature on FT in breast cancer patients from inception to 2022. Analysis of the annual volume of publications in this field revealed an overall upward trend in the global literature on FT of breast cancer in recent years. As many as 191 articles (31%) were published from 2020 to 2021, indicating the rapid development of research on FT of breast cancer and the increasing attention from clinical researchers.

Many countries/regions in the world have conducted research on FT of breast cancer, with the US in the leading position. Part of the supporting evidence shows that the top 5 publishers were all in the US, and 9 of the top 10 most productive authors were from the US. Moreover, articles published in the US had the highest average citation rates, which further demonstrates their strong academic influence. The reason may be that the concept of “financial toxicity” was first proposed by the American scholar Yousuf Zafar,^[32]^ and the American Society of Clinical Oncology established the Cancer Treatment Cost Panel in 2009, which proposed the intervention guidelines for FT of cancer,^[33]^ resulting in more mature research and higher quality of literature in this area in the US. Secondly, the FT prevalence survey and the construction of screening tools in the US are gradually taking shape. In 2015, the American Society of Clinical Oncology proposed a scoring system for comprehensive evaluation of anti-tumor treatment options according to the net health benefit and drug price of tumor treatment options, to make the limited medical resources more reasonably allocated by accurately measuring the value of new treatment, providing a reference for FT research in other countries.^[34]^ In addition, in the US, the cost of cancer treatment drugs and corresponding adjuvant therapies has been rising year by year, and many drugs are self-paying. A study of breast cancer patients showed that 17% of patients had out-of-pocket costs of >$5000.^[35]^ At the same time, the consumption concept of the American people, the low personal asset reserves, and reduced income due to limited ability to work as a result of the disease further increase the FT of cancer, which makes the US pay more and more attention to research on FT.^[10]^

Impact factor and journal partition are important indicators to evaluate the quality of journals, and the journal partition can weaken the structural imbalance factors between disciplines and facilitate the comparison and evaluation of journals in different disciplines.^[36]^ In this study, 7 of the top 10 journals in terms of volume of articles were in Q1 or Q2, indicating the overall high quality of the current literature. Published and frequently cited papers also have great academic influence. Four of the top 10 highly cited articles were authored by Yabroff KR, the most productive author and a key figure in this field. A randomized controlled trial article published in 2004 referred to the higher disease burden in cancer survivors than in individuals without cancer and the need for increased attention to this population.^[37]^ A questionnaire survey in 2016 determined that working-age cancer survivors usually experienced material, psychological, and financial difficulties, reminding healthcare professionals of the psychological state of these patients.^[38]^ An article published in *JNCI Journal of the National Cancer Institute* in 2008 estimated the cost of care for cancer patients and informed the development of national cancer plans and policies.^[39]^ Another article cited 299 times proposed a measure of the economic burden of cancer, estimated and predicted the cancer burden in the US at that time, identified key areas of future work, and informed healthcare policymakers, healthcare systems, and employers to improve cancer survivor experience in the US.^[40]^

The keyword analysis of the literature revealed that the high-frequency keywords for FT of breast cancer mainly focused on the impact of financial burden, patient care, quality of life, and treatment, and the research hotspots were traceable. Cluster analysis of the keywords found that the research focus was mainly on the cost of breast cancer treatment, the effect of drug treatment, the side effects of treatment, and theoretical research. One study showed that 25% of women with breast cancer experienced FT, and 12% of patients with early-stage breast cancer still had medical debt 4 years after diagnosis.^[41]^ FT not only affects the mental health and quality of life of patients but even increases their mortality in severe cases.^[42]^ In addition, since breast cancer patients usually undergo a series of adjuvant treatments, it can cause a series of treatment side effects.^[13–15]^ For some specific symptoms, enhancing care and preventing and reducing complications can also reduce the harm of FT. Analysis of the timeline view and burst keywords indicated that the demographic research on FT in breast cancer patients and its influencing factors were highly emergent, making it a frontier term in this field. Studies have shown that female, younger age, lower baseline income, adjuvant therapy, and recent disease diagnosis were the most frequently reported factors associated with FT.^[43]^ According to bibliometric analysis, the experience, burden, and work management of breast cancer survivors have also received increasing attention in the last 5 years. The research has shown that the FT in breast cancer patients is a multidisciplinary and multi-faceted social hotpot that requires the cooperation of the whole society and the collaboration of multidisciplinary researchers to apply the research results to clinical practice, so as to benefit more patients.^[44]^

## 5. Limitations

This study used CiteSpace to analyze and present the research trends in the field of FT of breast cancer over the past 30 years by taking the literature retrieved from the WoSCC as the object of study, and the results have a reference value. However, some limitations still exist in this study. First, although the WoSCC is the most commonly used database in scientometrics research, our data were only obtained from WoSCC without searching other databases such as Embase or PubMed. Secondly, our data analysis used computer tools instead of manual selection, and the analysis of the content was presented in keyword fields with possible omissions and biases in the detection of details. In the future, it will be necessary to thoroughly outline the study designs of the included literature and conduct a comprehensive analysis of the full text. In addition, considering the large time span of the study and the lack of standardization process for keywords in the WoSCC, keyword omissions may have occurred.

## 6. Conclusions

This study conducted a bibliometric analysis of the literatures on FT in breast cancer patients over the past 30 years and revealed an overall upward trend in research in this field. The US is the absolute leader in this field, and the UK and Australia have also proven to be major research forces in this field with high publication rates and centrality. Strong collaborations found among many developed countries and prestigious institutions suggest that FT of breast cancer is attracting increasing attention. Current research hotspots in this field are the influencing factors of FT, post-treatment side effects, and quality of life. In general, there is a paucity of studies on FT of breast cancer, with a narrow scope and limited collaboration among authors. In the future, more collaboration among authors, institutions, and countries is needed to promote the further development of research in this field.

## Author contributions

**Conceptualization:** Hui Cheng, Lu Lin, Li Tian.

Data curation: Hui Cheng.

Formal analysis: Hui Cheng, Tingting Liu.

Funding acquisition: Li Tian.

Methodology: Lu Lin, Tingting Liu, Shaotong Wang, Yueyue Zhang, Li Tian.

Resources: Hui Cheng, Yueyue Zhang.

Software: Hui Cheng, Shaotong Wang.

Supervision: Li Tian.

Writing – original draft: Hui Cheng, Lu Lin, Li Tian.

Writing – review & editing: Lu Lin, Li Tian.
